# Effects of Endogenous Anti-Oxidative Components from Different Vegetable Oils on Their Oxidative Stability

**DOI:** 10.3390/foods12112273

**Published:** 2023-06-05

**Authors:** Yuchen Ma, Guangyi Wang, Zeyuan Deng, Bing Zhang, Hongyan Li

**Affiliations:** 1 State Key Laboratory of Food Science and Resources, Nanchang University, Nanchang 330047, Chinadengzy@ncu.edu.cn (Z.D.); zhangbingair@126.com (B.Z.); 2 Institute for Advanced Study, Nanchang University, Nanchang 330031, China

**Keywords:** vegetable oils, fatty acids, endogenous antioxidants, oxidative stability, Rancimat, accelerated oxidation

## Abstract

The effects of endogenous anti-oxidative components of ten common edible vegetable oils (palm olein, corn oil, rapeseed oil, soybean oil, perilla seed oil, high oleic sunflower oil, peanut oil, camellia oil, linseed oil, and sesame oil) on oxidation were explored in this research. The oxidation processes and patterns of the oils were investigated with the Schaal oven test using fatty acids and the oxidative stability index, acid value, peroxide value, *p*-anisidine value, total oxidation value, and content of major endogenous anti-oxidative components as indicators. The major endogenous anti-oxidative components in vegetable oils were tocopherols, sterols, polyphenols, and squalene, among which α-tocopherol, β-sitosterol, and polyphenols showed good anti-oxidative activity. However, squalene and polyphenols were relatively low and showed limited anti-oxidative effects. Moreover, the oxidative stability index of edible vegetable oils oxidized at high temperature (120 °C) was positively correlated with the content of saturated fatty acids (*r* = 0.659) and negatively correlated with the content of polyunsaturated fatty acids (*r* = −0.634) and calculated oxidizability (*r* = −0.696). When oxidized at a low temperature (62 °C), oxidative stability was influenced by a combination of fatty acid composition as well as endogenous anti-oxidative components. An improved TOPSIS based on Mahalanobis distance was used to evaluate the oxidative stability of different types of vegetable oils. Moreover, the oxidative stability of corn oil was better than the other vegetable oils, while perilla seed oil was very weak.

## 1. Introduction

Vegetable oil is an important source of energy and fatty acid intake for humans. The oxidation of oils produces a large number of free radicals and carbonyl compounds, which can lead to oxidation and degradation reactions of amino acids and proteins [1]. Carbonyl compounds can cause nucleophilic degradation of food components, which is an important factor that damages the quality and safety of food [2], while fat oxidation is one of the most important sources of carbonyl compounds in food [3]. Numerous applications of the oxidative stability of oils exist. The use of biofuels to increase the oxidative stability of oils has been the subject of numerous investigations. Moudden et al. [4] extracted polyphenols from the ground-up waste of olives to lengthen the shelf life of canola and sunflower oils. Polyphenols were isolated from walnut shells by Zahra et al. [5] to increase the oxidative stability of biodiesel.

For different vegetable oils, the fatty acid composition and the type and content of trace components such as tocopherols and sterols are diverse. For example, palm olein and coconut oil are rich in saturated fatty acids (SFAs) [6,7], while soybean oil, peanut oil, rapeseed oil, and sunflower oil mainly contain unsaturated fatty acids (UFAs) [8]. In addition to fatty acids, sunflower oil, peanut oil, palm oil, and camellia oil have higher α-tocopherol content, while rapeseed oil and perilla seed oil mainly contain γ-tocopherol [8]. Corn oil and rapeseed oil have higher phytosterol content than soybean oil, sunflower oil, and camellia oil, with camellia oil having the lowest phytosterol content [9].

Tocopherol is a kind of trace component with high anti-oxidative activity, and the type of tocopherol contained in different vegetable oils are different. Sunflower oil, corn oil, peanut oil, camellia oil, and palm olein mainly contain α-tocopherol. Perilla oil and canola oil mainly contain γ-tocopherol, and soybean oil mainly contains δ-tocopherol [10,11]. Réblová et al. [12] found that the anti-oxidative activities of α-T and δ-T were different at different temperatures, with δ-T activity being about twice that of α-T at 80 °C and similar at 130 °C. Our previous study [8] also found that the oxidative degradation of α-tocopherol was faster in oils with high linoleic acid content when the linolenic acid content was low. Tocopherol’s anti-oxidative impact has two components. One is to neutralize free radicals in the system as a free radical scavenger, and the other is to quench singlet oxygen as an oxygen scavenger in order to prevent the chain initiation and chain propagation stages of the lipid oxidation cycle [13].

The most extensively researched phenolic compounds, i.e., prunetin and quercetin, exhibit considerable anti-oxidative activity in addition to tocopherols. The capacity of quercetin to scavenge highly reactive free radicals may be related to its potential positive health effects [14]. The anti-oxidative mechanism of phenolic compounds may be due to the free radical scavenging ability of hydroxyl groups and the ability of phenolic compounds themselves to chelate metals and inhibit oxidative enzymes [15,16].

Vegetable oils are also abundant in β-sitosterol, stigmasterol, and squalene, all of which have potent inhibitory effects on the oxidation of lipids. A concentration-dependent, anti-oxidative activity of squalene was observed during the heat-induced oxidation of olive oil [17]. Regarding squalene, studies on the free radical oxidation of squalene led to different perspectives. Early studies identified cyclic dihydroperoxides as the main oxidation product [18], whereas more recent studies reported that alcohols and epoxides were mainly formed [19].

The majority of recent investigations on endogenous antioxidants in vegetable oils have concentrated on a particular antioxidant activity (Table 1). Few studies have examined the binary interactions of endogenous antioxidants in intricate oil matrices. The outcomes of solely chemical solvent media cannot accurately reflect the interplay of endogenous antioxidants in vegetable oils [20]. Moreover, it is incredibly challenging to thoroughly analyze the antioxidant processes of endogenous antioxidants utilizing oil as a matrix due to the intricacy of vegetable oil matrices.

Vegetable oil’s primary endogenous antioxidant components experience considerable alterations during oxidation. In order to understand how endogenous antioxidants affect the oxidative stability of vegetable oils in complex oil matrices, it is necessary to first perform a thorough examination of these changes, which is performed in this work. The purpose of this study was to ascertain the function of vegetable oils’ endogenous antioxidant components in the oxidation process by tracking changes in the acid value, peroxide value, *p*-anisidine value, and content of endogenous anti-oxidative components during the accelerated oxidation of vegetable oils. Additionally, the correlation among the oxidation indicators was removed using an improved TOPSIS approach [25], creating a theoretical foundation for figuring out the oxidative stability of vegetable oils.

## 2. Materials and Methods

### 2.1. Materials

Ten edible vegetable oils not containing synthetic antioxidants were purchased at a supermarket in Nanchang, Jiangxi. Palm olein was obtained from Julong Group Co., Ltd. (Tianjin, China). Corn oil and soybean oil were obtained from Yihai Kerry Arawana Holdings Co., Ltd. (Shanghai, China). Rapeseed oil was obtained from Standard Foods (Shanghai, China). Perilla seed oil was obtained from Xin qi Canon (Xingtai, China). High oleic sunflower oil was obtained from Nisshin (Shanghai, China). Peanut oil was obtained from Luhua Group Co., Ltd. (Laiyan, China). Camellia oil was obtained from Jinhao Camellia Oil Co., Ltd. (Changsha, China). Linseed oil was obtained from Hongjingyuan (Inner Mongolia, China). Sesame oil was obtained from Sancheng Oil Industry Co., Ltd. (Nanyang, China).

### 2.2. Standards and Chemicals

Standard fatty acid methyl ester (GLC-463) was purchased from Nu-Chek Prep Inc. (Elysian, MN, America). Tocopherol, phytosterol, squalene, and polyphenol standards were purchased from Beijing Solarbio Science & Technoligy Co., Ltd. (Beijing, China). Segetalin-A was purchased from ShanghaiyuanyeBio-Technology Co., Ltd. (Shanghai, China). Methanol, n-hexane, acetonitrile, and isopropanol were purchased from Merck (Darmstadt, Germany).

### 2.3. Analysis of Fatty Acids

According to the method described by Zou et al. [26], the oil sample was transformed into fatty acid methyl esters. An Agilent 6890N gas chromatographic column connected with an FID detector and a CP-Sil 88-fused silica capillary column (CP7489, 100 m × 0.25 mm × 0.2 m, Agilent) were applied to investigate the treated samples. With a sample volume of 10 μL, a constant pressure splitless injection was used. Hydrogen (99.99%) served as the carrier gas, and the column pressure was 24.52 psi. The temperature in the column was held constant at 45 °C for 4 min, then increased from 45 °C to 175 °C at a rate of 13 °C/min for 27 min, and then increased from 215 °C at a rate of 4 °C/min for 35 min. The FID was 250 °C, with nitrogen (99.99%) serving as the fuel gas and flowing at a rate of 30 mL/min along with hydrogen and air, respectively, at 30 mL/min and 300 mL/min.

### 2.4. Rancimat Measurements

Using 743 Rancimat, the oxidative stability index (OSI) was calculated using the method described in ISO 6886: 2016. The oil sample (2–3 g) was heated to 120 °C with 10 L/h of air forced into it, and the generated volatiles were injected into water before the oil’s oxidative stability was tested by monitoring the inflection point of the change in water conductivity.

### 2.5. Oxidation Products

According to the approved AOCS method [27,28], the acid value (method Cd 3d-63) and hydrogen peroxide content (method Cd 8b-90) were ascertained. According to the procedure outlined in ISO 6885: 2016, the *p*-anisidine value and total oxidation value were computed and determined.

### 2.6. Analysis of Tocopherols

The tocopherol content of the vegetable oil samples was determined according to the method described by Ahmed et al. [29] with slight modifications. Vegetable oil (1.0 g) was fixed to 10 mL with n-hexane. Agilent 1260 HPLC was used to detect the sample, which had been filtered with a 0.45 μm filter using an Elite Hypersil ODS2 column (5 μm, 4.6 mm × 150 mm) with a VWD detector. With a flow rate of 0.3 mL/min, n-hexane/isopropanol (99.3:0.7, *v*/*v*) served as the mobile phase. The detection wavelength was 295 nm, and the temperature of the column was 30 °C.

### 2.7. Analysis of Phytosterol and Squalene

The pretreatment of the oil samples was performed using the method of Zhang et al. [30] and Shi et al. [31]. Oil samples weighing 0.25 g were saponified, and the unsaponifiables were extracted for a 1 h derivatization reaction. Gas chromatography in series with mass spectrometry (GC-MS) with a DB-5 MS capillary column (0.25 mm, 30 m × 0.25 mm) was used to evaluate the treated samples. The following setup parameters were used: split injection ratio of 50:1, flow rate of 1.2 mL/min of helium, transfer line temperature of 250 °C, ion source temperature of 230 °C, and injector temperature of 280 °C. After holding the column temperature at 200 °C for 0.5 min, it was increased to 300 °C at a rate of 10 °C/min and maintained at this temperature for 20 min. The modified EI ionization mode ranged from 50 to 650.

### 2.8. Total Phenolic Content

By slightly modifying the method described by Ahmed et al. [29], the total phenolic content of the vegetable oils was ascertained. The extractions were combined after being performed three times on an oil sample (2.0 g) using 1 mL of hexane and 2 mL of 80% methanol. After 3 min of reacting 1 mL of the extract with water (2 mL) and FC reagent (0.5 mL), 1.5 mL of a 1% sodium carbonate solution was added. The absorbance at 765 nm was measured after one hour. Additionally, the outcomes were reported as mg gallic acid equivalent (GAE)/kg oil.

### 2.9. Determination of Cyclolinopeptides

The qualitative and quantitative analysis of cyclolinopeptides was evaluated using the method of Zou et al. [32], where 20 μL of internal standard Seg-A (1.0 mg/mL) and 1 mL of n-hexane were applied to 1.5 g of the oil sample. With the help of a SPE silica gel column, the combined solution was separated. The eluent contained 13 mL of 100% n-hexane, 13 mL of n-hexane solution of 20% (*v*/*v*) ethyl acetate (EtOAc), 13 mL of hexane solution of 50% (*v*/*v*) EtOAc, 13 mL of 100% EtOAc, and 13 mL of dichloromethane solution of 10% (*v*/*v*) methanol. [33] The last two eluent fractions were recovered and concentrated at 40 °C in a vacuum. The concentrated sample was dissolved in methanol and put through a 0.22 μm PTFE syringe filter. After that, the sample was identified using a mass spectrometer (6538 Accurate-Mass QTOF LC/MS system, Agilent) fitted with an orthogonal electrospray ionization (ESI) source [34]. The compounds were ionized in positive ion mode, and MS spectra were gathered from m/z 100 to m/z 1500. The following system settings were used: 4.0 kV capillary voltage, 350 °C drying gas temperature, 135 V fragmentor voltage, 10.0 L/min drying gas flow rate, 40 psi nebulizing gas pressure, and 45 eV collision energy. The Agilent 1100 series HPLC system was outfitted with a DAD and a Zorbax Eclipse XDB-C18 column (4.6 mm × 250 mm × 5 µm, Agilent). Acetonitrile and deionized water served as the mobile phases. The following elution conditions were used: 0 to 25 min, 45 to 65% acetonitrile; 25 to 45 min, isocratic holding at 65% acetonitrile; and 45 to 50 min, 65 to 45% acetonitrile. A flow rate of 0.8 mL/min at 30 °C was used for the column temperature. Peaks at 214 nm (10 nm bandwidth) were measured in comparison to a 300 nm reference signal. A 0.22 μm PTFE syringe filter with a sample capacity of 10 μL was used to filter the methanol-solubilized material.

### 2.10. Schaal Oven Test

In accordance with the procedure outlined by Cao et al. [8], vegetable oil (300 g) was put in glass bottles with caps, gently capped, and then placed in a constant-temperature oven at 62 °C for 25 days with continuous heating and oxidation. The bottles were shaken every 12 h, with the positions changing. Every five days, the right amount of oxidized oil was taken for evaluation and analysis.

### 2.11. Evaluating the Oxidative Stability of Vegetable Oils Using Improved TOPSIS

The specific steps in the improved TOPSIS method were as follows [25].

Assume there are *m* possible outcomes (A_1_, A_2_, ……, A_m_) and *n* decision indices (B_1_, B_2_, ……, B_n_). Let X_ij_ stand for the value of A_i_’s criteria on B_j_ (i = 1, 2, …, m; j = 1, 2, …, n). The decision matrix X was established (x_ij_), as shown in Table S3.

Following that, the ideal solution S^+^ and the negative ideal solution S^−^ are identified using Equation (1):

(1) $$S^{+}=\left\{s_{1}^{+},s_{2}^{+},\cdots ,s_{n}^{+}\right\},\;S^{-}=\left\{s_{1}^{-},s_{2}^{-},\cdots ,s_{n}^{-}\right\}.$$

The benefit index B_j_ is determined using Equation (2):

(2) $$s_{j}^{+}=\max\left\{x_{\mathrm{ij}}|1\ll i\ll m\right\},\;s_{j}^{-}=\min\left\{x_{\mathrm{ij}}|1\ll i\ll m\right\}.$$

The cost index B_j_ is determined using Equation (3):

(3) $$s_{j}^{+}=\min\left\{x_{\mathrm{ij}}|1\ll i\ll m\right\},\;s_{j}^{-}=\max\left\{x_{\mathrm{ij}}|1\ll i\ll m\right\}.$$

For each alternative, the Mahalanobis distance from the ideal and non-ideal solutions is calculated. The distance between alternative A_i_ and the constructive ideal solution is calculated according to Equation (4):

(4) $$d_{i}^{+}=\sqrt{\left(x_{\mathrm{ij}}-s_{j}^{+}\right)Y^{-1}{\left(x_{\mathrm{ij}}-s_{j}^{+}\right)}^{\prime }},\;i=1,2,\cdots ,m.$$

Alternative A_i_’s distance from the non-ideal solution is calculated according to Equation (5):

(5) $$d_{i}^{-}=\sqrt{\left(x_{\mathrm{ij}}-s_{j}^{-}\right)Y^{-1}{\left(x_{\mathrm{ij}}-s_{j}^{-}\right)}^{\prime }},\;i=1,2,\cdots ,m.$$

Of note is that Y is the covariance matrix of X. When Y is the unit matrix, the Mahalanobis distance would degenerate into the Euclidean distance.

Finally, we calculate the relative closeness of each alternative to the ideal solution according to Equation (6):

(6) $$Q_{i}=\frac{d_{i}^{-}}{d_{i}^{-}+d_{i}^{+}},\;i=1,2,\cdots ,m.$$

The alternatives are ranked based on their relative closeness. A_i_ is the superior alternative if Q_i_ is higher, and vice versa.

### 2.12. Statistical Analysis

Three parallel measurements of the samples were made, and the results were represented as mean ± standard deviation. Using a one-way ANOVA, statistical differences were identified using Duncan’s technique. *p*-values less than 0.05 denote significant differences between data. All data were analyzed and compared using SPSS 26.0 software, with graphs created using Origin 2019b. Matlab 16.0 was used to create the improved TOPSIS.

## 3. Results and Discussion

### 3.1. The Oxidative Stability Index

**Figure 1:**
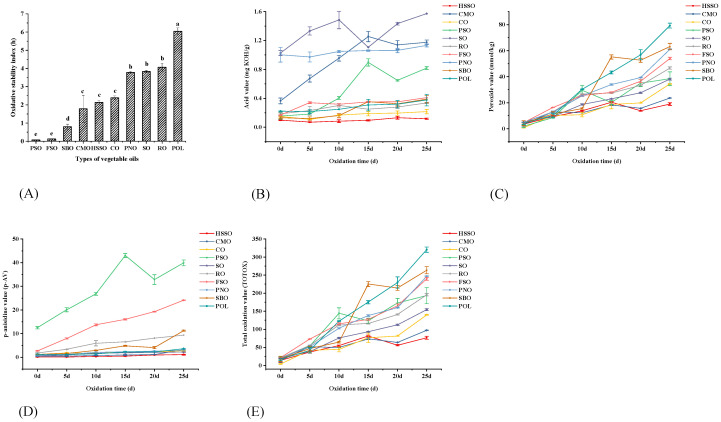
Changes in oxidation indicators during the accelerated oxidation of vegetable oils. Different lower-case letters indicate significant differences (*p* ≤ 0.05) between the different vegetable oils; POL: palm olein; CO: corn oil; PSO: perilla seed oil; PNO: peanut oil; CMO: camellia seed oil; RO: rapeseed oil; SBO: soybean oil; HSSO: high oleic acid sunflower seed oil; FSO: flaxseed oil; SO: sesame oil. (**A**) Changes in the oxidative stability index (120 °C); (**B**) changes in the acid value (mg KOH/g); (**C**) changes in the peroxide value (mmol/kg); (**D**) changes in the *p*-anisidine value; and (**E**) changes in the total oxidation value.

The Rancimat method, a conductometric method for accelerated oxidation assessment that measures the oxidation induction time to measure stability, is frequently used in studies to test the oxidative stability of oils [35]. As shown in Figure 1A, the oxidative stability index of flaxseed oil (FSO) and perilla seed oil (PSO) was lower at 120 °C and 10 L/h, followed by soybean oil (SBO), camellia oil (CMO), high oleic sunflower seed oil (HSSO), corn oil (CO), peanut oil (PNO), sesame oil (SO), rapeseed oil (RO), and palm olein (POL). The oxidative stability index results showed that the order of the oxidation stability of vegetable oils was POL > RO > SO > PNO > CO > HSSO > CMO > SBO > FSO > PSO. According to the research of Li et al. [36], the oxidation amount of soybean oil under Rancimat circumstances at the induction time point was unaffected by the temperature or duration of heating. As a result, it appears that the level of oxidation at induction is particularly important and may hold the key to connecting Rancimat data with the oil’s stability at ambient temperature.

As can be seen from Table 2, the oxidative stability index was positively correlated with the content of SFA and negatively correlated with the content of PUFA and calculated oxidizability (COX) under the condition that only the difference in fatty acid composition was considered. Multari et al. [37] discovered that soybean oil with a high linoleic acid concentration (52.9%) displayed a fatty acid structure that was vulnerable to oxidation by examining the changes in fatty acid composition and oxidation products of vegetable oils with varied fatty acid compositions following short-term frying. The results in Table 2 are in agreement with this.

### 3.2. Chemical Quality Analysis

#### 3.2.1. Analysis of Fatty Acids

**Figure 2:**
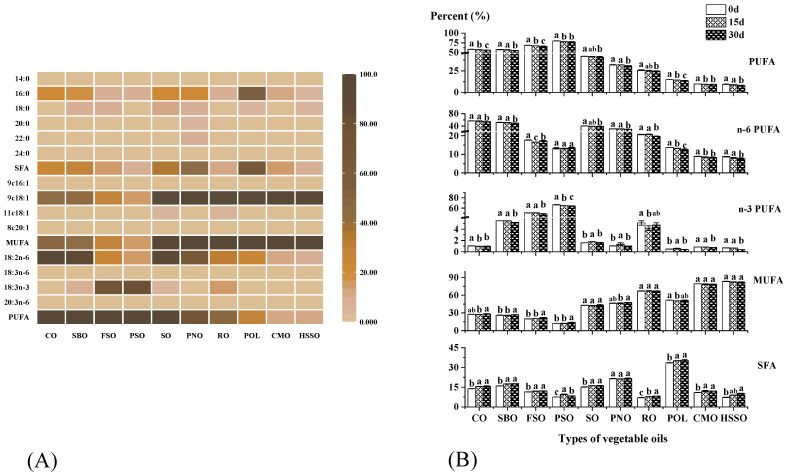
(**A**) The initial fatty acid composition of the ten vegetable oil samples. (**B**) Changes in the fatty acid composition of oils during the accelerated oxidation test (n = 3). Mean values with standard deviations are plotted as bars; different lower-case letters indicate significant differences (*p* ≤ 0.05) in vegetable oils at different oxidation times; POL: palm olein; CO: corn oil; PSO: perilla seed oil; PNO: peanut oil; CMO: camellia seed oil; RO: rapeseed oil; SBO: soybean oil; HSSO: high oleic acid sunflower seed oil; FSO: flaxseed oil; SO: sesame oil; PUFA: polyunsaturated fatty acid; n-6 PUFA: n-6 polyunsaturated fatty acid; n-3 PUFA: n-3 polyunsaturated fatty acid; MUFA: monounsaturated fatty acid; SFA: saturated fatty acid.

The composition of fatty acids is closely related to the oxidative stability of vegetable oils. The initial fatty acid composition of ten vegetable oil samples is presented in Figure 2A. The results showed that the monounsaturated fatty acid (MUFA) content of POL was 51.69%. The saturated fatty acid (SFA) content (33.54%) of POL was significantly lower than that (≥40%) of commercially available palm oil, and the same conclusion was found by Rossi et al. [38]. PSO and FSO mainly contained α-linolenic acid (50.91–66.54%), while the rest of the vegetable oils mainly contained oleic acid and linoleic acid (63.45–88.69%). Polyunsaturated fatty acids (PUFAs) were the most significant elements in the oxidation process of vegetable oils [8]. Therefore, PSO and FSO with high PUFA content are particularly susceptible to oxidation, which was also verified using our subsequent measurements.

The SFA content of the samples ranged from 7.12% to 33.54%, the MUFA content ranged from 12.56% to 83.28%, and the PUFA content ranged from 9.47% to 79.94%. POL and PNO had higher SFAs than other oils. HSSO, CMO, RO, POL, PNO, and SO had a higher monounsaturated fatty acid content. PSO and FSO had a higher PUFA content. As shown in Figure 2B, PUFA, n-6PUFA, and n-3PUFA decreased with the increase in oxidation days. n-6PUFA decreased more significantly in corn oil and soybean oil (2.06% and 2.33%, respectively), and n-3PUFA decreased more in flaxseed oil and perilla seed oil (2.42% and 2.34%, respectively). The percentages of SFA and MUFA increased, with CO and SBO increasing more in SFAs by 1.73% and 1.59%, respectively, and FSO and PSO increasing more in MUFAs by 1.73% and 1.54%, respectively.

#### 3.2.2. Oxidation Products

##### Acid Value

As shown in Figure 1B, the acid values of SO and PNO before oxidation were higher than those of the other edible vegetable oils, reaching 1.03 and 1.00 mg KOH/g, respectively. After 25 days of oxidation using oven heating, the acid values of all ten vegetable oils increased, with the acid values of PSO, CMO, SBO, POL, SO, and FSO rising more significantly, by 0.66, 0.81, 0.26, 0.15, 0.54, and 0.24 mg KOH/g, respectively. The acid values for the remaining four vegetable oils were only slightly elevated. At the end of oxidation, each vegetable oil was ranked from highest to lowest acid value as SO (1.57), CMO (1.18), PNO (1.13), PSO (0.82), FSO (0.41), SBO (0.39), POL (0.38), RO (0.34), and CO (0.22). Although the acid value showed an overall tendency to increase during oxidation, the range of variation was not large except for PSO, CMO, and SO. The AV of vegetable oils, as measured by Cao et al. [8], was found to increase consistently with the number of storage days, but the relative increase was also not significant. Although the acid value is an indicator for hydrolytic deterioration, there is not a large range of variation in the acid value of edible oils with limited moisture and oxygen content and low-temperature oxidation.

##### Peroxide Value (POV)

The amount of hydroperoxides, the primary product of oil oxidation, is generally expressed as the peroxide value (POV), which is the most widely used method to evaluate the degree of oxidation of oils. As shown in Figure 1C, the POV of vegetable oils increased with the increase in accelerated oxidation time in the oven, and the POV of PSO and POL increased quickly from 0 to 10 days of oxidation, by 27.48 and 26.33 mmol/kg, respectively. From 10 to 20 days of oxidation, the POV of SBO and POL increased faster than the other edible vegetable oils, by 37.15 and 26.49 mmol/kg, respectively. The POV of POL and PNO increased more rapidly than the other edible vegetable oils from 20 days to the end of oxidation, with an increase of 22.39 and 21.73 mmol/kg, respectively.

The order of POV at the end of oxidation was POL > SBO > PNO > FSO > RO > PSO > SO > CO > CMO > HSSO. Among them, the POV of PSO, CMO, and HSSO had fluctuating changes, probably because the oxidation of oil to produce peroxide is accompanied by secondary oxidation reactions, and the peroxides break down to form secondary oxidation products such as aldehydes, ketones, and epoxy resins. It is speculated in the study by Cao et al. [8] that POV may also drop abruptly when the decomposition rate of peroxide is greater than the formation rate, resulting in a fluctuating pattern of variation.

##### *p*-Anisidine Value

As shown in Figure 1D, the *p*-anisidine values of edible vegetable oils increased with the increase in oxidation time, among which the initial *p*-anisidine value of PSO was the highest, reaching 12.51. From 0 to 10 days of oxidation, the *p*-anisidine values of PSO and FSO increased the fastest, by 14.33 and 10.97, respectively, while those of RO increased by 4.09. The *p*-anisidine values of HSSO, CMO, CO, and POL were stable or even slightly decreased. From 10 to 20 days of oxidation, PSO and FSO increased by 6.04 and 5.57, respectively. From 20 days of oxidation to the end of oxidation, SBO increased by 7.18, PSO increased by 7.03, and FSO, CMO, CO and POL also showed high increases. The order of *p*-anisidine values at the end of oxidation was PSO > FSO > SBO > RO > POL > CMO > PNO > CO > SO > HSSO.

The total oxidation value has the advantage of incorporating indicators for both primary oxidation products (hydroperoxides) and secondary oxidation products (unsaturated aldehydes), which is useful when determining the extent of oxidative degradation of lipids [39,40,41]. As shown in Figure 1E, the total oxidation value of PSO increased most significantly from 0 to 10 days of oxidation, and POL, FSO, RO, and PNO increased more significantly. The total oxidation value of SBO and POL increased most significantly from 10 to 20 days of oxidation. POL, PNO, FSO, and CO increased more significantly from 20 days to the end of oxidation. The order of total oxidation values at the end of oxidation was POL > SBO > PNO > FSO > RO > PSO > SO > CO > CMO > HSSO.

Table 2 shows a significant positive correlation between TOTOX and the content of SFA (*r* = 0.720) but no significant correlation with either MUFA or PUFA. This is significantly different from the correlation between OSI and fatty acid composition. This is due to the complexity of the oxidation process of vegetable oils, which is influenced by other factors such as endogenous anti-oxidative components in addition to the fatty acid composition. By measuring the content of endogenous anti-oxidative components in vegetable oils during oxidation, the oxidation process and pattern of oils can be further studied and analyzed [8].

### 3.3. Endogenous Antioxidants

#### 3.3.1. Tocopherols

**Figure 3:**
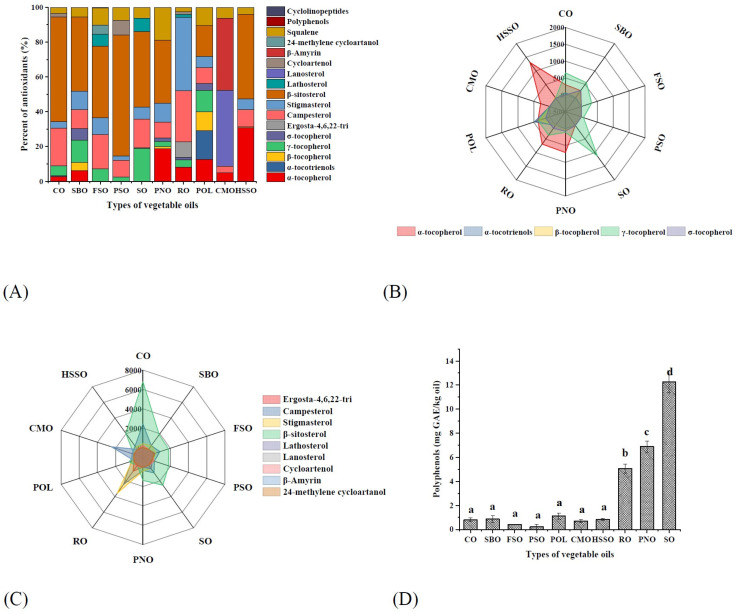
Composition of endogenous antioxidants in different vegetable oils. Different lower-case letters indicate significant differences (*p* ≤ 0.05) between the different vegetable oils; POL: palm olein; CO: corn oil; PSO: perilla seed oil; PNO: peanut oil; CMO: camellia seed oil; RO: rapeseed oil; SBO: soybean oil; HSSO: high oleic acid sunflower seed oil; FSO: flaxseed oil; SO: sesame oil. (**A**) Percent of endogenous antioxidants; (**B**) tocopherol isomer content (mg/kg); and (**C**) phytosterol content (mg/kg); (**D**) polyphenol content (mg GAE/kg oil).

As seen in Figure 3A, there were significant differences in the composition and content of endogenous anti-oxidative components contained in different vegetable oils. α-tocopherol, γ-tocopherol, β-sitosterol, stigmasterol, and campesterol were the main components in the vegetable oils. CMO mainly contains lanosterol and β-amyrin. Moreover, these 10 vegetable oils mainly contained α-tocopherol and γ-tocopherol (Figure 3B), among which PNO, RO, HSSO, CMO, and POL were dominated by α-tocopherol (284–1306 mg/kg), and the remaining 5 vegetable oils were dominated by γ-tocopherol (67–1122 mg/kg). In addition, POL also had a high α-tocotrienol content (478 mg/kg), while SBO, PNO, HSSO, and POL contained small amounts of β-tocopherol (22–318 mg/kg). SBO, PNO, RO, and POL also contained small amounts of σ-tocopherol (75–284 mg/kg).

**Figure 4:**
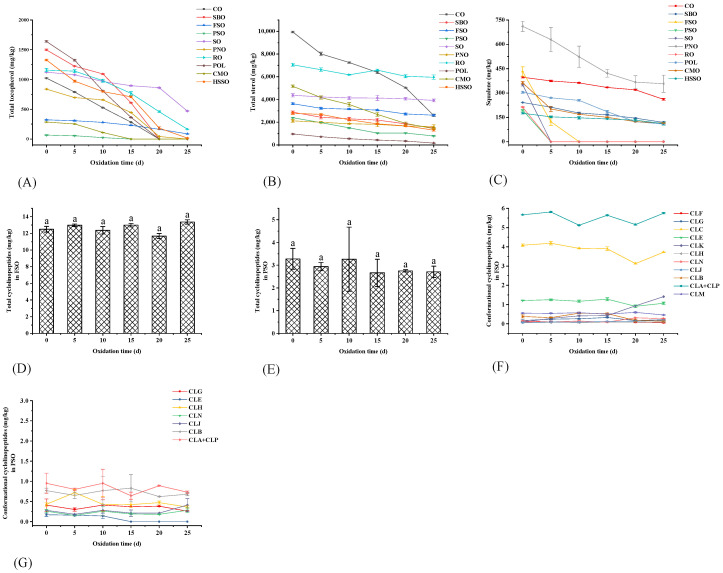
Changes in the content of endogenous anti-oxidative components during accelerated oxidation. Different lower-case letters indicate significant differences (*p* ≤ 0.05) in vegetable oils at different oxidation times; POL: palm olein; CO: corn oil; PSO: perilla seed oil; PNO: peanut oil; CMO: camellia seed oil; RO: rapeseed oil; SBO: soybean oil; HSSO: high oleic acid sunflower seed oil; FSO: flaxseed oil; SO: sesame oil. (**A**) Changes in total tocopherols; (**B**) changes in total sterols; (**C**) changes in squalene; (**D**) changes in total cyclolinopeptides in FSO; (**E**) changes in total cyclolinopeptides in PSO; (**F**) changes in different conformational cyclolinopeptides in FSO; and (**G**) changes in different conformational cyclolinopeptides in PSO.

The total tocopherols in vegetable oils showed a gradual decrease throughout the oxidation process (Figure 4A). Before accelerated oxidation in the oven, the levels of total tocopherols in these 10 vegetable oils were: POL > SBO > HSSO > RO > SO > CO > PNO > FSO > CMO > PSO. Compared to other vegetable oils, CMO and PSO contained fewer total tocopherols, and they were depleted by the 15th day of oxidation. POL, SBO, and CO were depleted by the 20th day of oxidation, while HSSO still contained a small number of tocopherols on the 25th day. From the changes in total tocopherol content, the accelerated oxidation of vegetable oils showed a sharp decrease in total tocopherol content by days 10–20. This is because, in the process of accelerated oxidation, the hydrogen atoms provided by tocopherol can form stable oxidation products with the free radicals produced by oil oxidation, which can protect the oil from oxidation while being oxidized [42]. In the early stage of accelerated oxidation (0–5 d), the oxidation of tocopherols was relatively slow. When the accelerated oxidation proceeded to the middle and late stages (10–20 d), the content of tocopherols in vegetable oils decreased sharply. This is because, as the rancidity of the vegetable oil increases, its free radical content increases, thus consuming a large number of tocopherols in the vegetable oil.

**Figure 5:**
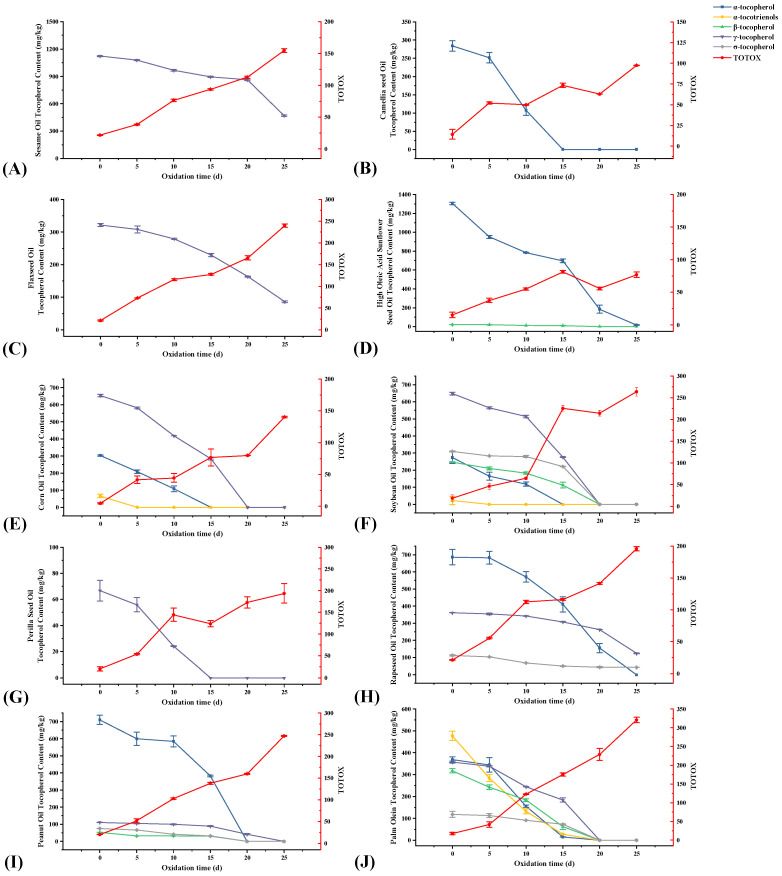
Changes in tocopherol compounds and total oxidation value during the accelerated oxidation test (n = 3). Mean values with standard deviations are plotted as lines. (**A**) Sesame oil; (**B**) camellia seed oil; (**C**) flaxseed oil; (**D**) high oleic acid sunflower seed oil; (**E**) corn oil; (**F**) soybean oil; (**G**) perilla seed oil; (**H**) rapeseed oil; (**I**) peanut oil; and (**J**) palm olein.

Karmowski et al. [43] determined the anti-oxidative activity of tocopherols and tocotrienols at different concentrations and showed that their anti-oxidative activities were in the following order: α-tocotrienol > α-tocopherol > β-tocotrienol ≈ β-tocopherol > γ-tocotrienol > γ-tocopherol > σ-tocotrienol > σ-tocopherol. As seen in Figure 5, after 10 days of oxidation, the percentage loss of α-tocotrienol and α-tocopherol from POL were higher than those of the other conformations of tocopherols, at 72.3% and 57.7%, respectively, while the percentage loss of β-tocopherol, γ-tocopherol, and σ-tocopherol decreased in the order of 42.2%, 31.5%, and 23.2%, respectively. The percentage loss of different conformations of tocopherols from SBO after 10 days of oxidation had the same ranking: α-tocotrienol (100%) > α-tocopherol (56.9%) > β-tocotrienol (25.9%) > γ-tocopherol (20.7%) > σ-tocopherol (9.6%). However, the highest percentage loss of σ-tocopherol was found in PNO and RO on day 10 of oxidation, reaching 44.5% and 40.07%, respectively. This occurrence in PNO may be due to the low initial content of σ-tocopherol (75.24 mg/kg). Although the percentage loss of σ-tocopherol was the highest in RO during the first 15 days of oxidation, that of different conformations of tocopherols at the end of oxidation was in the following order: α-tocopherol (100%) > γ-tocopherol (66.0%) > σ-tocopherol (62.6%).

#### 3.3.2. Phytosterol

From Figure 3C, the phytosterols in these 10 vegetable oils were mainly campesterol, stigmasterol, and β-sitosterol, among which β-sitosterol was dominant. Among these 10 edible vegetable oils, only CMO and RO do not contain β-sitosterol. In addition, CO, RO, and PSO also contained cycloartenol; RO and FSO contained lathosterol; RO contained its unique ergosta-4,6,22-tri; FSO contained its unique 24-methylene cycloartenol; and CMO contained its unique lanosterol and β-amyrin. Before the accelerated oxidation, the contents of phytosterols in these 10 vegetable oils were: CO > RO > CMO > SO > FSO > SBO > HSSO > PSO > PNO > POL (Figure 4B). After accelerated oxidation in the oven, the phytosterols all showed different degrees of degradation. This is a result of their simplicity in formation and the corresponding allylic free radicals’ relative stability [44].

**Figure 6:**
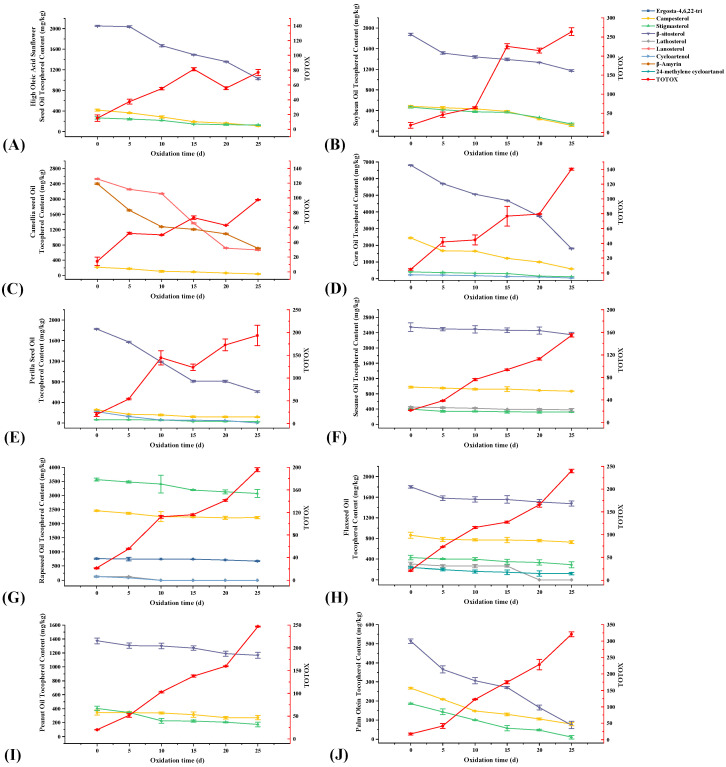
Changes in phytosterols and total oxidation value during the accelerated oxidation test (n = 3). Mean values with standard deviations are plotted as lines. (**A**) High oleic acid sunflower seed oil; (**B**) soybean oil; (**C**) camellia seed oil; (**D**) corn oil; (**E**) perilla seed oil; (**F**) sesame oil; (**G**) rapeseed oil; (**H**) flaxseed Oil; (**I**) peanut oil; and (**J**) palm olein.

During the accelerated oxidation, the phytosterol degradation content in SO was the least with a content reduction of 446 mg/kg, followed by POL with a reduction of 510 mg/kg. The content in CO degraded the most significantly with a reduction of 7347 mg/kg. However, POL had the highest percentage loss of phytosterols, reaching 83.0% (Figure S1). As shown in Figure 6, the degradation of β-sitosterol, lanosterol, and β-amyrin was obvious, and the loss of β-sitosterol at the end of oxidation was 1025, 705, 4992, 1213, 325, 206, 440, and 195 mg/kg for HSSO, SBO, CO, PSO, FSO, PNO, POL, and SO, respectively. The loss contents of lanosterol and β-amyrin from CMO were 1862 and 1691 mg/kg, respectively.

The phytosterols in vegetable oils have some anti-oxidative effects. The Rancimat method and the DPPH radical scavenging experiment were used by Liu et al. [20] to assess the antioxidant effect of phytosterols in the rice bran oil matrix. They discovered that phytosterols had no discernible antioxidant activity under the Rancimat test conditions and actually had a pro-oxidant effect. It has been demonstrated that hotter environments encourage the oxidation of phytosterols, resulting in oxidation products that lessen the antioxidant action of phytosterols [22,45]. Since these phytosterols were more effective at lower temperatures (60 °C) with longer times than at higher temperatures (120 °C and 180 °C) with shorter times, Chen et al. [22] showed that their anti-oxidative effectiveness was temperature- and time-dependent.

#### 3.3.3. Squalene

As shown in Figure 4C, before oxidation, the levels of squalene were PNO > FSO > CO > CMO > SO > POL > SBO > RO > PSO > HSSO. The percentage losses from RO, SO, FSO, and PSO reached 100% on days 5–10 of oxidation. The final percentage loss of squalene from CMO was 68.4%, but it already reached 44.5% on day 5 of oxidation. In POL, squalene degradation occurred mainly on days 10–20 of oxidation, with the percentage loss increasing from 16.4% to 59.5%. There was no significant change in the squalene degradation rate during the oxidation of SBO, CO, PNO, and HSSO (Figure S1).

It was found that the content and type of tocopherols play an important role in the oxidative degradation of squalene. Among the 10 edible vegetable oils, only RO, SO, and FSO did not reach 100% loss of tocopherols at the end of oxidation with 85.8%, 58.2%, and 73.3%, respectively. Tocopherols in PSO, although lost at 100%, had a low initial content of 66.67 mg/kg. In contrast, these four edible vegetable oils were completely degraded by squalene at the initial stage of oxidation (0–10 d). After the complete degradation of squalene in RO, FSO, and PSO, their tocopherol degradation rates increased (Supplementary Figure S1). This suggested that squalene exerted a significant anti-oxidative effect when the level of tocopherols was low or its anti-oxidative effect was poor.

Less research has been performed on the antioxidant interactions between squalene and tocopherols. Psomiadou et al. [17] hypothesized that squalene might regenerate α-tocopherol in photooxidation tests, which is in line with Kohno et al. [46]. The Crocin bleaching method was used by Finotti et al. [47] to analyze the antioxidant interactions between α-tocopherol, β-sitosterol, and squalene. The results demonstrated that squalene works in synergy with α-tocopherol and β-sitosterol. This might be because squalene can act as a competing substance in the reaction, slowing down oxidation.

#### 3.3.4. Polyphenols

Virgin vegetable oils undergo different degrees of refining processes before becoming commercially available commercial vegetable oils, which leads to another reduction in trace amounts of polyphenols in the oil. As shown in Figure 3D, the polyphenol content in these 10 vegetable oils ranged from 0.22 to 12.26 mg GAE/kg oil, with SO having the highest polyphenol content (12.26 mg GAE/kg oil), followed by PNO (6.88 mg GAE/kg oil) and RO (5.07 mg GAE/kg oil), respectively, and the remaining seven kinds of oils having a lower polyphenol content. The higher polyphenol content in SO was related to its higher content of lignans. Phenolic substances in vegetable oils play a major anti-oxidative role in the initial stage of oxidation of edible vegetable oils, but the content of phenolic substances in commercially available vegetable oils is low. In addition, polyphenols are susceptible to oxidative degradation during accelerated oxidation, which results in their undetectable content after 5 days of accelerated oxidation. Therefore, although polyphenols have high anti-oxidative activity [14], refined edible vegetable oils are almost always low in polyphenols, which results in a very limited effect of polyphenols on the oxidation process of edible vegetable oils.

#### 3.3.5. Cyclolinopeptides

Twelve conformations of cyclolinopeptides were identified in FSO, and eight conformations of cyclolinopeptides were identified in PSO for the first time. As shown in Figure 4D–G, the types of cyclolinopeptides in FSO were more than those in PSO, and the total cyclolinopeptides content in FSO (12.5 mg/kg) was higher than that in PSO (3.28 mg/kg). The low cyclolinopeptides content was due to the fact that the cyclolinopeptides contained in the refined FSO were greatly reduced after refining the oil, and the vast majority were lost in the refining process, such as degumming and decolorization. Zeng et al. [48] provided guidance on the retention of more cyclic peptides in the refining process using a study on the comparative effects of different oil extraction methods on the nutrient composition in flaxseed oil.

During the accelerated oxidation in the oven, there was no significant difference in the changes in total cyclolinopeptide contents in FSO and PSO, but the cyclolinopeptides of different conformations would be interconverted with each other. From Figure 4F, it can be seen that during the accelerated oxidation process, the content of [1–9-NαC]-linusorb B2 (CLB) increased and then decreased, the content of [1–9-NαC], [1-MetO]-linusorb B2 (CLC) decreased and then increased, and the content of [1–9-NαC], [1-MetO_2_]-linusorb B2 (CLK) gradually increased. There was a small decrease in the content of CLE, and a small increase in the content of [1–8-NαC], [1-MetO_2_]-linusorb B1 (CLJ). Since the content of cyclolinopeptides was low in the measured refined FSO, the trends in various conformations could not be clearly shown. Zou et al. [32] showed that during the oxidation of FSO, cyclolinopeptides containing methionine Met were quickly oxidized to cyclolinopeptides of methionine sulfoxide Mso and methionine sulfone Msn. Therefore, cyclolinopeptides have some anti-oxidative activity, and they may act as antioxidants by chelating metal ions or other mechanisms. The small amount of cyclolinopeptides in PSO has not been reported in the literature, so its role in the oxidation process of PSO needs to be further investigated.

### 3.4. Evaluation of the Oxidative Stability of Vegetable Oils

From the previous results, the fatty acid composition and endogenous anti-oxidative components of edible vegetable oils jointly influence their oxidation process. To comprehensively evaluate the oxidative stability of vegetable oils, the improved TOPSIS method [25] with OSI, and the amount of change in fatty acid composition and endogenous antioxidant components as indicators, were used. The linear correlations among different indicators are shown in Table 3, and some of them showed high relations. Among them, the correlation coefficients between SFA and squalene, POV and TOTOX, campesterol and β-sitosterol, campesterol and total sterols, and β-sitosterol and total sterols exceeded 0.80, reaching a significant level (*p* < 0.01). The improved TOPSIS is appropriate for taking into account the correlations among the indicators, which is in line with the characteristics of the oxidative stability assessment of vegetable oils. Therefore, the improved TOPSIS method was used in this section to evaluate the oxidative stability of vegetable oils.

The closeness of the ten vegetable oils is shown in Table 4. From Table 4, the oxidative stability of the ten vegetable oils in comprehensive consideration was as follows: CO > PNO > SO > HSSO > FSO > CMO > POL > SBO > RO > PSO. Among them, the closeness of PSO is much lower than that of the other vegetable oils. Instead of using the Euclidean distance as in the traditional TOPSIS method, the improved TOPSIS method used the Mahalanobis distance. Although this enhancement reduced the impact of correlation between indicators to some degree, dealing with the situation of multiple indicators with strong connections is more challenging. The complexity was increased by the variety of endogenous antioxidants in vegetable oils and their interactions, as well as the variety of oxidation indicators. However, the majority of the present evaluation techniques concentrate on the interrelationship between indicator weights or the use of a single information indicator, making it challenging to take both completeness and correlation into account at the same time [25]. The evaluation process needs to be improved with a more precise and succinct mechanism that can extract the evaluation results from the entire layout.

## 4. Conclusions and Perspective

The oxidative stability of edible vegetable oils was influenced by both fatty acid composition and endogenous anti-oxidative components. The endogenous anti-oxidative components in vegetable oils were mainly tocopherols, sterols, polyphenols, and squalene. Tocopherols were mainly α-tocopherol and γ-tocopherol, and sterols were mainly campesterol, stigmasterol, and β-sitosterol. Among them, α-tocopherol, β-sitosterol, and polyphenols showed high anti-oxidative activity, and squalene and polyphenols contained relatively little and had limited effects. Small amounts of cyclolinopeptides were also present in FSO (12.5 mg/kg) and PSO (3.28 mg/kg), and the overall amount was stable following oxidation with conformational conversion. In addition, significant interactions existed between tocopherols and squalene. At low tocopherol concentrations, squalene’s antioxidant effects are more evident. According to a thorough analysis using the TOPSIS method, PSO had lower oxidative stability than the other vegetable oils, and CO had the best oxidative stability among the evaluated vegetable oils.

The role of endogenous antioxidant elements in the intricate oxidation process of vegetable oils was examined in this work. Additionally, the TOPSIS method was used to analyze the connections between various oxidation indicators and to assess the oxidative stability of various kinds of vegetable oils. As a result, it offered evidence demonstrating how antioxidants can be combined to lengthen the shelf life of the oil. Vegetable oil components interact with one another in intricate ways that sometimes cross over. As a result, this study was unable to determine in-depth connections between antioxidant effects and the content and type of endogenous antioxidant components, respectively. Based on the results of this study, it would be necessary for future studies to investigate the various endogenous antioxidants added to vegetable oil matrices in compound combinations of various concentrations and species, respectively, in order to further analyze the multivariate interactions between various combinations of endogenous antioxidant components and to provide a more specific basis for exploring endogenous antioxidants to retard the oxidation of vegetable oils.

## Figures and Tables

**Table 1:** Studies on the antioxidant effect of endogenous antioxidants in vegetable oils.

Antioxidant	Method	System	Result	Reference
Tocopherols	Schaal oven test assay	Vegetable oils	At high quantities, tocopherols promote oxidation while acting as antioxidants at low concentrations. Additionally, different vegetable oils have different inflection points in terms of concentration.	Cao et al., 2015 [8]
Quercetin	Schaal oven test assay	Camelina oil	When quercetin is dissolved in camellia oil, phospholipids increase its antioxidant power and solubility.	Maria et al., 2021 [21]
Phytosterols	(1) DPPH radical scavenging assay; (2) Hydroxyl radical scavenging assay; (3) β-Carotene protection assay; (4) Accelerated oxidation of soybean oil.	Ethanol solution and soybean oil	Compared to non-lipid systems, lipid systems have a higher capacity for antioxidants in phytosterols. Furthermore, it works better in colder conditions than it does in hotter ones.	Chen et al., 2019 [22]
Tocopherol and tocotrienol	Chemometric methods	Vegetable oils	Tocopherol type and content are largely controlled by the kind of vegetable oil and are not always correlated with the level of oil refinement.	Wen et al., 2020 [23]
Tocopherol, polyphenol, and phytosterol	Free radical scavenging capacity (ABTS, DPPH and FRAP assay)	Methanol	The key elements influencing the antioxidant potential of the studied seed oils were polyphenols, brassicasterol, and α-tocopherol.	Yao et al., 2019 [24]
α-tocopherol and phytosterol	(1) Rancimat test; (2) DPPH free radical scavenging assay.	Purified rice bran oil	Compared to phytosterol, α-tocopherols has stronger antioxidant effect. Additionally, they have negative impacts on the oil matrix.	Liu et al., 2021 [20]

**Table 2:** Correlation analysis between the oxidative stability index, oxidation products, and fatty acid composition of vegetable oil.

Index	OSI	ΔAV	ΔPOV	Δ*p*-AV	ΔTOTOX	SFA%	MUFA%	PUFA%	COX
OSI	1								
ΔAV	−0.333	1							
ΔPOV	0.369	−0.323	1						
Δ*p*-AV	−0.675 *	0.321	0.126	1					
ΔTOTOX	0.275	−0.275	0.992 **	0.250	1				
SFA%	0.659 *	−0.245	0.783 **	−0.352	0.720 *	1			
MUFA%	0.436	−0.054	−0.420	−0.680 *	−0.496	−0.063	1		
PUFA%	−0.634 *	0.131	0.154	0.772 **	0.248	−0.261	−0.947 **	1	
COX	−0.696 *	0.257	0.077	0.944 **	0.194	−0.354	−0.833 **	0.920 **	1

Note: * and ** mean significant at the 0.05 level (bilateral) and significant at the 0.01 level (bilateral); OSI: oxidative stability index; Δ: increment of data; SFA: saturated fatty acid; MUFA: monounsaturated fatty acid; PUFA: polyunsaturated fatty acid; COX: [1 × (C18:1) + 10.3 × (C18:2) + 21.6 × (C18:3)]/100^8^.

**Table 3:** The results of the correlation test.

Index	OSI	ΔSFA	ΔMUFA	ΔPUFA	ΔAV	ΔPOV	Δ*p*-AV	ΔTOTOX	Δα-Tocopherol	Δγ-Tocopherol	ΔTocopherol	ΔCampesterol	Δβ-Sitosterol	ΔStigmasterol	ΔTotal Sterol	ΔSqualene	ΔPolyphenols
OSI	1.00																
ΔSFA	0.07	1.00															
ΔMUFA	−0.48	−0.71 *	1.00														
ΔPUFA	0.65 *	−0.06	−0.66 *	1.00													
ΔAV	−0.33	−0.38	0.11	0.25	1.00												
ΔPOV	0.37	−0.30	0.39	−0.16	−0.32	1.00											
Δ*p*-AV	−0.68 *	−0.38	0.75 *	−0.66 *	0.32	0.13	1.00										
ΔTOTOX	0.28	−0.35	0.48	−0.24	−0.28	0.99 **	0.25	1.00									
Δα-Tocopherol	−0.30	−0.54	0.64 *	−0.31	0.59	0.24	0.52	0.30	1.00								
Δγ-Tocopherol	−0.16	−0.06	−0.19	0.29	0.19	−0.32	0.21	−0.29	−0.42	1.00							
ΔTocopherol	−0.57	−0.57	0.46	−0.10	0.71 *	−0.43	0.60	−0.35	0.53	0.37	1.00						
ΔCampesterol	0.05	−0.34	0.01	0.36	0.35	0.17	0.27	0.20	0.00	0.49	0.20	1.00					
Δβ-Sitosterol	0.12	−0.32	−0.07	0.47	0.34	0.19	0.12	0.20	−0.02	0.41	0.12	0.97 **	1.00				
ΔStigmasterol	0.03	−0.18	−0.22	0.46	0.58	−0.45	0.21	−0.42	0.08	0.55	0.59	0.49	0.45	1.00			
ΔTotal sterol	0.19	−0.27	0.09	0.18	0.01	0.38	0.22	0.39	−0.08	0.23	−0.07	0.90 **	0.86 **	0.24	1.00		
ΔSqualene	−0.01	0.82 **	−0.51	−0.14	−0.25	−0.22	−0.24	−0.25	−0.49	−0.04	−0.54	−0.42	−0.44	−0.24	−0.37	1.00	
ΔPolyphenols	−0.48	0.41	0.02	−0.46	−0.08	0.01	0.35	0.05	−0.06	0.30	−0.02	−0.26	−0.29	−0.18	−0.39	0.47	1.00

Note: * and ** mean significant at the 0.05 level (bilateral) and significant at the 0.01 level (bilateral); Δ: increment of data.

**Table 4:** The closeness of the ten vegetable oils evaluated with the improved TOPSIS.

			Improved TOPSIS	
Oils	d^+^	d^−^	Closeness	Order
CO	6.379	5.350	0.456	1
SBO	8.778	4.728	0.350	8
FSO	7.644	5.309	0.410	5
PSO	9.177	1.738	0.159	10
SO	7.072	5.344	0.430	3
PNO	6.663	5.141	0.436	2
RO	8.466	4.362	0.340	9
POL	8.215	4.773	0.368	7
CMO	7.878	5.260	0.400	6
HSSO	7.068	5.052	0.417	4

## Data Availability

The data presented in this study are available on request from the corresponding authors.

## Supplementary Materials

The following supporting information can be downloaded at: https://www.mdpi.com/article/10.3390/foods12112273/s1, Figure S1: Changes in the percentage losses of endogenous antioxidant components from different edible vegetable oils during the accelerated oxidation test; Figure S2: HPLC-VWD chromatograms at 295 nm for tocopherols in ten edible vegetable oils during oxidation; Figure S3: The total ion chromatogram (TIC) for the seven phytosterol standards; Figure S4: The total ion chromatogram (TIC) for the ten edible vegetable oils; Figure S5: The results for the identification of cyclolinopeptides using HPLC-ESI-QTOF-MS; Table S1: The results for the identification of squalene and sterol in ten edible vegetable oils using GC-MS; Table S2: Cyclolinopeptides in flaxseed oil detected using HPLC-QTOF-MS; Table S3: Decision matrix for the improved TOPSIS.

## Abbreviations and Nomenclatures Used

POL, palm olein; RO, rapeseed oil; SO, sesame oil; PNO, peanut oil; CO, corn oil; HSSO, high oleic sunflower oil; CMO, camellia oil; SBO, soybean oil; FSO, linseed oil; PSO, perilla seed oil; CNO, coconut oil; OSI, oxidative stability index; SFA, saturated fatty acid; MUFA, monounsaturated fatty acid; PUFA, polyunsaturated fatty acid; n-6 PUFA, n-6 Polyunsaturated fatty acid; n-3 PUFA, n-3 Polyunsaturated fatty acid; COX, calculated oxidizability; AV, acid value; POV, peroxide value; *p*-AV, *p*-anisidine value; TOTOX, total oxidation value; CLA, [1-9-NαC]-linusorb B3; CLB, [1–9-NαC]-linusorb B2; CLC, [1–9-NαC], [1-MetO]-linusorb B2; CLE, [1–8-NαC], [1-MetO]-linusorb B1; CLF, [1-8-NαC], [1,3-MetO]-linusorb A3; CLG, [1–8-NαC], [1,3-MetO]-linusorb A1; CLH, [1–8-NαC], [1-MetO]-linusorb A1; CLJ, [1–8-NαC], [1-MetO_2_]-linusorb B1; CLK, [1–9-NαC], [1-MetO_2_]-linusorb B2; CLM, [1–8-NαC]-linusorb A1; CLN, [1–8-NαC], [3-MetO]-linusorb A1; CLP, [1-8-NαC]-linusorb B1.

## References

[1] Hidalgo F.J., Zamora R. (2016). Amino Acid Degradations Produced by Lipid Oxidation Products. Crit. Rev. Food Sci. Nutr..

[2] Zamora R., Navarro J.L., Aguilar I., Hidalgo F.J. (2015). Lipid-derived aldehyde degradation under thermal conditions. Food Chem..

[3] Ganesan B., Brothersen C., McMahon D.J. (2014). Fortification of Foods with Omega-3 Polyunsaturated Fatty Acids. Crit. Rev. Food Sci. Nutr..

[4] El Moudden H., El Idrissi Y., Belmaghraoui W., Belhoussaine O., El Guezzane C., Bouayoun T., Harhar H., Tabyaoui M. (2020). Olive mill wastewater polyphenol-based extract as a vegetable oil shelf life extending additive. J. Food Process. Preserv..

[5] Khounani Z., Hosseinzadeh-Bandbafha H., Nizami A.-S., Sulaiman A., Goli S.A.H., Tavassoli-Kafrani E., Ghaffari A., Rajaeifar M.A., Kim K.-H., Talebi A.F. (2020). Unlocking the potential of walnut husk extract in the production of waste cooking oil-based biodiesel. Renew. Sustain. Energy Rev..

[6] Padial-Jaudenes M., Castanys-Munoz E., Ramirez M., Lasekan J. (2020). Physiological Impact of Palm Olein or Palm Oil in Infant Formulas: A Review of Clinical Evidence. Nutrients.

[7] Deen A., Visvanathan R., Wickramarachchi D., Marikkar N., Nammi S., Jayawardana B.C., Liyanage R. (2021). Chemical composition and health benefits of coconut oil: An overview. J. Sci. Food Agric..

[8] Cao J., Li H., Xia X., Zou X.-G., Li J., Zhu X.-M., Deng Z.-Y. (2015). Effect of fatty acid and tocopherol on oxidative stability of vegetable oils with limited air. Int. J. Food Prop..

[9] Shi T., Zhu M., Zhou X., Huo X., Long Y., Zeng X., Chen Y. (2019). H-1 NMR combined with PLS for the rapid determination of squalene and sterols in vegetable oils. Food Chem..

[10] Karabulut I., Topcu A., Yorulmaz A., Tekin A., Ozay D.S. (2005). Effects of the industrial refining process on some properties of hazelnut oil. Eur. J. Lipid Sci. Technol..

[11] Pagani M.A., Baltanás M.A. (2010). Production of natural antioxidants from vegetable oil deodorizer distillates: Effect of catalytic hydrogenation. Bioresour. Technol..

[12] Réblová Z. (2006). The effect of temperature on the antioxidant activity of tocopherols. Eur. J. Lipid Sci. Technol..

[13] Liu R., Xu Y., Zhang T., Gong M., Liu R., Chang M., Wang X. (2022). Interactions between liposoluble antioxidants: A critical review. Food Res. Int..

[14] Rather R.A., Bhagat M. (2020). Quercetin as an innovative therapeutic tool for cancer chemoprevention: Molecular mechanisms and implications in human health. Cancer Med..

[15] Araujo F.F., Farias D.P., Neri-Numa I.A., Pastore G.M. (2021). Polyphenols and their applications: An approach in food chemistry and innovation potential. Food Chem..

[16] Munekata P.E.S., Nieto G., Pateiro M., Lorenzo J.M. (2020). Phenolic Compounds Obtained from Olea europaea By-Products and Their Use to Improve the Quality and Shelf Life of Meat and Meat Products-A Review. Antioxidants.

[17] Psomiadou E., Tsimidou M. (1999). On the role of squalene in olive oil stability. J. Agric. Food Chem..

[18] Bolland J.L., Hughes H. (1949). The primary thermal oxidation product of squalene. J. Chem. Soc..

[19] Naziri E., Consonni R., Tsimidou M.Z. (2014). Squalene oxidation products: Monitoring the formation, characterisation and pro-oxidant activity. Eur. J. Lipid Sci. Technol..

[20] Liu R., Xu Y., Chang M., Tang L., Lu M., Liu R., Jin Q., Wang X. (2021). Antioxidant interaction of alpha-tocopherol, gamma-oryzanol and phytosterol in rice bran oil. Food Chem..

[21] del Pilar Garcia-Mendoza M., Espinosa-Pardo F.A., Savoire R., Harscoat-Schiavo C., Cansell M., Subra-Paternault P. (2021). Improvement of the oxidative stability of camelina oil by enrichment with phospholipid-quercetin formulations. Food Chem..

[22] Chen J., Tang G., Zhou J., Liu W., Bi Y. (2019). The characterization of soybean germ oil and the antioxidative activity of its phytosterols. Rsc. Adv..

[23] Wen Y., Xu L., Xue C., Jiang X., Wei Z. (2020). Assessing the impact of oil types and grades on tocopherol and tocotrienol contents in vegetable oils with chemometric methods. Molecules.

[24] Yao Y., Liu W., Zhou H., Zhang D., Li R., Li C., Wang S. (2019). The relations between minor components and antioxidant capacity of five fruits and vegetables seed oils in China. J. Oleo Sci..

[25] Wang Z.-X., Wang Y.-Y. (2014). Evaluation of the provincial competitiveness of the Chinese high-tech industry using an improved TOPSIS method. Expert Syst. Appl..

[26] Zou X.-G., Hu J.-N., Zhao M.-L., Zhu X.-M., Li H.-Y., Liu X.-R., Liu R., Deng Z.-Y. (2014). Lipozyme RM IM-Catalyzed Acidolysis of *Cinnamomum camphora* Seed Oil with Oleic Acid To Produce Human Milk Fat Substitutes Enriched in Medium-Chain Fatty Acids. J. Agric. Food Chem..

[27] AOCS (2017). Acid Value of fats and oils. AOCS Official Method Cd 3d-63.

[28] AOCS (2011). Peroxide value acetic acid-isooctane method, official methods and recommended practices of the AOCS. AOCS Official Method Cd 8b-90.

[29] Ahmed I.A.M., Uslu N., Ozcan M.M., Juhaimi F.A.L., Ghafoor K., Babiker E.E., Osman M.A., Alqah H.A.S. (2021). Effect of conventional oven roasting treatment on the physicochemical quality attributes of sesame seeds obtained from different locations. Food Chem..

[30] Zhang T., Wang T., Liu R., Chang M., Jin Q., Wang X. (2020). Chemical characterization of fourteen kinds of novel edible oils: A comparative study using chemometrics. LWT.

[31] Shi T., Wu G., Jin Q., Wang X. (2021). Detection of camellia oil adulteration using chemometrics based on fatty acids GC fingerprints and phytosterols GC–MS fingerprints. Food Chem..

[32] Zou X.-G., Hu J.-N., Zhu X.-M., Wang Y.-F., Deng Z.-Y. (2018). Methionine sulfone-containing orbitides, good indicators to evaluate oxidation process of flaxseed oil. Food Chem..

[33] Gui B., Shim Y.Y., Datla R.S.S., Covello P.S., Stone S.L., Reaney M.J.T. (2012). Identification and Quantification of Cyclolinopeptides in Five Flaxseed Cultivars. J. Agric. Food Chem..

[34] Zou X.-G., Chen X.-L., Hu J.-N., Wang Y.-F., Gong D.-M., Zhu X.-M., Deng Z.-Y. (2017). Comparisons of proximate compositions, fatty acids profile and micronutrients between fiber and oil flaxseeds (*Linum usitatissimum* L.). J. Food Compos. Anal..

[35] Yang K.-M., Hsu F.-L., Chen C.-W., Hsu C.-L., Cheng M.-C. (2018). Quality characterization and oxidative stability of camellia seed oils produced with different roasting temperatures. J. Oleo Sci..

[36] Li X., Li Y., Yang F., Liu R., Zhao C., Jin Q., Wang X. (2019). Oxidation degree of soybean oil at induction time point under Rancimat test condition: Theoretical derivation and experimental observation. Food Res. Int..

[37] Multari S., Marsol-Vall A., Heponiemi P., Suomela J.-P., Yang B. (2019). Changes in the volatile profile, fatty acid composition and other markers of lipid oxidation of six different vegetable oils during short-term deep-frying. Food Res. Int..

[38] Rossi M., Alamprese C., Ratti S. (2007). Tocopherols and tocotrienols as free radical-scavengers in refined vegetable oils and their stability during deep-fat frying. Food Chem..

[39] Hyatt J.R., Zhang S., Akoh C.C. (2021). Comparison of antioxidant activities of selected phenolic compounds in O/W emulsions and bulk oil. Food Chem..

[40] Ahmadian F., Aminzare M., Mohseni M., Hoseini M., Hassanzadazar H. (2022). Eugenol and Clove (*Syzygium aromaticum*) Essential Oil Efficacy on Oxidative Stability of Sunflower Oil during Accelerated Storage. J. Med. Plants By-Prod..

[41] Saleh F., Al-Otaibi M.M., Al-Zoreky N. (2021). Quality Assessment of Frying Oil from some Restaurants in Al Ahsa, Saudi Arabia. Sci. J. King Faisal Univ..

[42] Azzi A. (2021). Reflections on a century of vitamin E research: Looking at the past with an eye on the future. Free Radic. Biol. Med..

[43] Karmowski J., Hintze V., Kschonsek J., Killenberg M., Boehm V. (2015). Antioxidant activities of tocopherols/tocotrienols and lipophilic antioxidant capacity of wheat, vegetable oils, milk and milk cream by using photochemiluminescence. Food Chem..

[44] Ramadan M.F., Moersel J.-T. (2006). Screening of the antiradical action of vegetable oils. J. Food Compos. Anal..

[45] Winkler J.K., Warner K. (2008). The effect of phytosterol concentration on oxidative stability and thermal polymerization of heated oils. Eur. J. Lipid Sci. Technol..

[46] Kohno Y., Egawa Y., Itoh S., Nagaoka S.-I., Takahashi M., Mukai K. (1995). Kinetic study of quenching reaction of singlet oxygen and scavenging reaction of free radical by squalene in n-butanol. Biochim. Biophys. Acta (BBA)-Lipids Lipid Metab..

[47] Finotti E., D’ambrosio M., Paoletti F., Vivanti V., Quaglia G. (2000). Synergistic effects of α-tocopherol, β-sitosterol and squalene on antioxidant activity assayed by crocin bleaching method. Nahrung.

[48] Zeng J., Xiao T., Ni X., Wei T., Liu X., Deng Z.-Y., Li J. (2022). The comparative analysis of different oil extraction methods based on the quality of flaxseed oil. J. Food Compos. Anal..

