# Supplementary material for: Effects of Endogenous Anti-Oxidative Components from Different Vegetable Oils on Their Oxidative Stability
Source: Foods. 2023 Jun 5;12(11):2273. doi: 10.3390/foods12112273 (PMC10252449; doi:10.3390/foods12112273)
Supplement: Supplementary file 1 [file foods-12-02273-s001.zip › foods-2410483-supplementary.pdf]

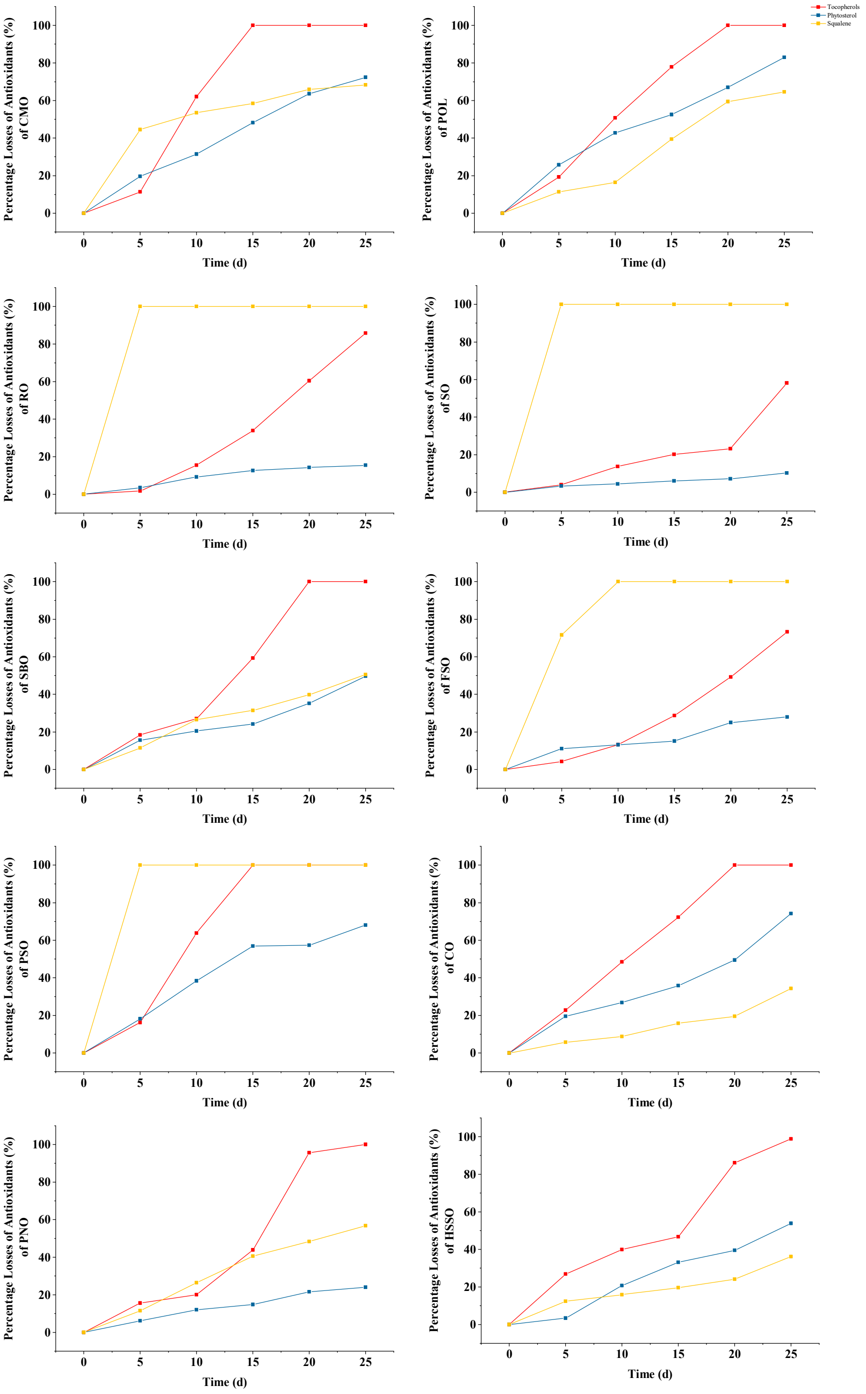

Figure S1. Changes of the percentage losses of endogenous antioxidant components in different edible vegetable oils during accelerated oxidation test. POL: Palm olein; CO: Corn Oil; PSO: Perilla Seed Oil; PNO: Peanut Oil; CMO: Camellia seed Oil; RO: Rapeseed oil; SBO: Soybean Oil; HSSO: High Oleic Acid Sunflower Seed Oil; FSO: Flaxseed Oil; SO: Sesame Oil.

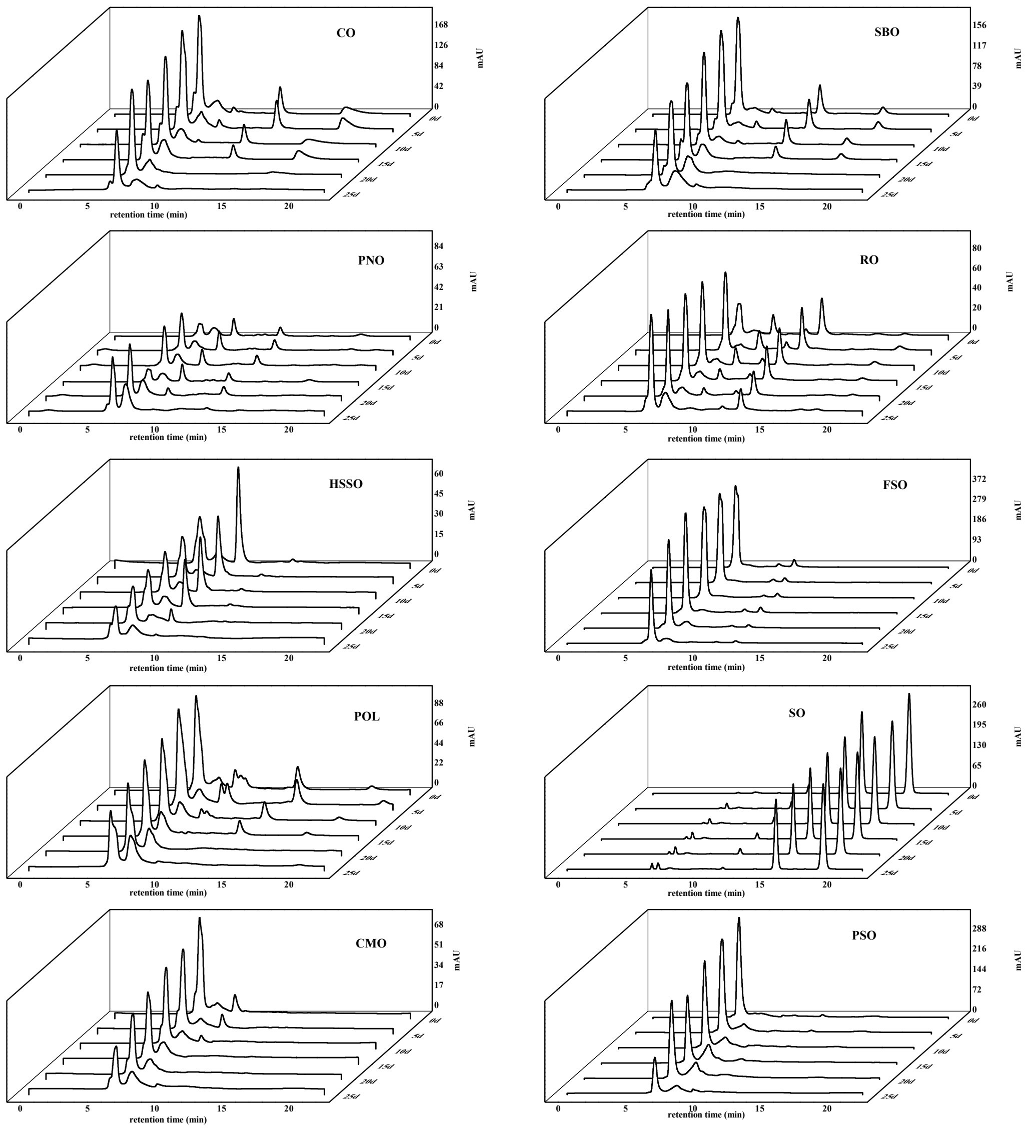

Figure S2. HPLC-VWD chromatograms of at 295 nm for tocopherols in ten edible vegetable oils during oxidation. POL: Palm olein; CO: Corn Oil; PSO: Perilla Seed Oil; PNO: Peanut Oil; CMO: Camellia seed Oil; RO: Rapeseed oil; SBO: Soybean Oil; HSSO: High Oleic Acid Sunflower Seed Oil; FSO: Flaxseed Oil; SO: Sesame Oil.

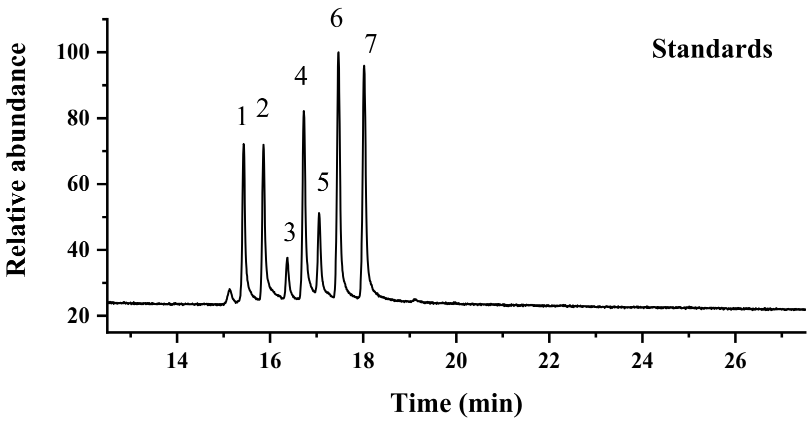

Figure S3. The total ion chromatogram (TIC) of seven phytosterol standards. 1- Campesterol; 2-Stigmasterol; 3- Ergosta-4,6,22-tri; 4-β-sitosterol; 5- Lanosterol; 6-β-Amyrin; 7- Cycloartenol.

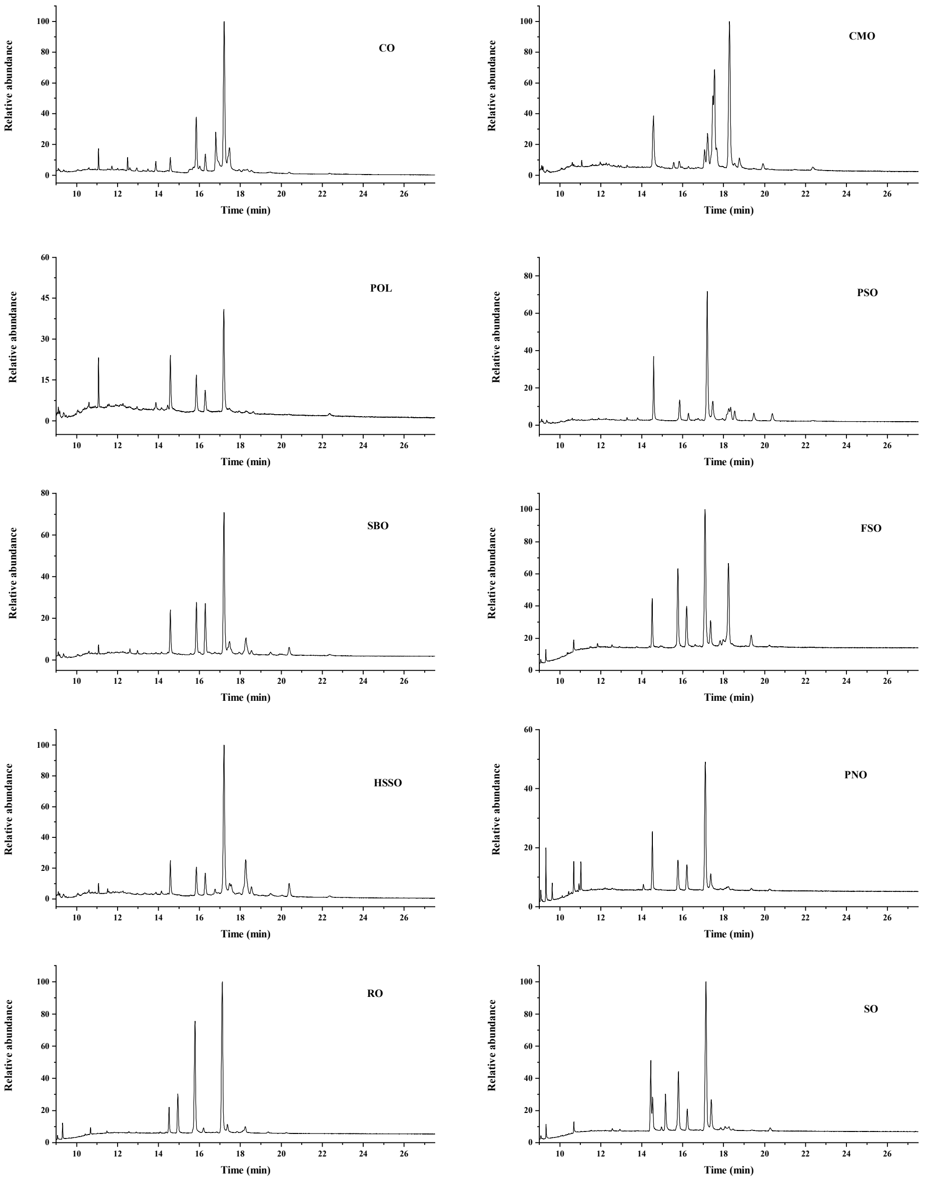

Figure S4. The total ion chromatogram (TIC) in ten edible vegetable oils. POL: Palm olein; CO: Corn Oil; PSO: Perilla Seed Oil; PNO: Peanut Oil; CMO: Camellia seed Oil; RO: Rapeseed oil; SBO: Soybean Oil; HSSO: High Oleic Acid Sunflower Seed Oil; FSO: Flaxseed Oil; SO: Sesame Oil.

Table S1. The results of identification of squalene and sterol in ten edible vegetable oils by GC-MS.

Peak No.	Retention time (min)	Compound	Chemical formula	$[M+H]^+$	Fragment ions(m/z)
1	11.077	Squalene	$C_{30}H_{50}O$	69	55, 81
2	14.584	5α-Cholestan-3β-ol	$C_{30}H_{56}O$	215	75, 445
3	14.966	Ergosta-4,6,22-triene	$C_{28}H_{44}$	380	255, 431
4	15.854	Campesterol	$C_{28}H_{48}O$	382	367, 472
5	16.286	Stigmasterol	$C_{29}H_{48}O$	394	379, 484
6	17.218	β-sitosterol	$C_{32}H_{58}O$	396	381, 482
6	17.397	Lathosterol	$C_{27}H_{46}O$	386	371, 394
7	17.477	Lanosterol	$C_{33}H_{58}O$	393	368, 483
8	17.573	β-Amyrin	$C_{33}H_{58}O$	218	203, 257
9	18.372	9,19-Cyclolanost-24-en-3-ol	$C_{33}H_{58}O$	365	339, 393
10	19.395	24-Methylene-9,19-cyclolanostan-3β-ol	$C_{31}H_{52}O$	422	380, 451

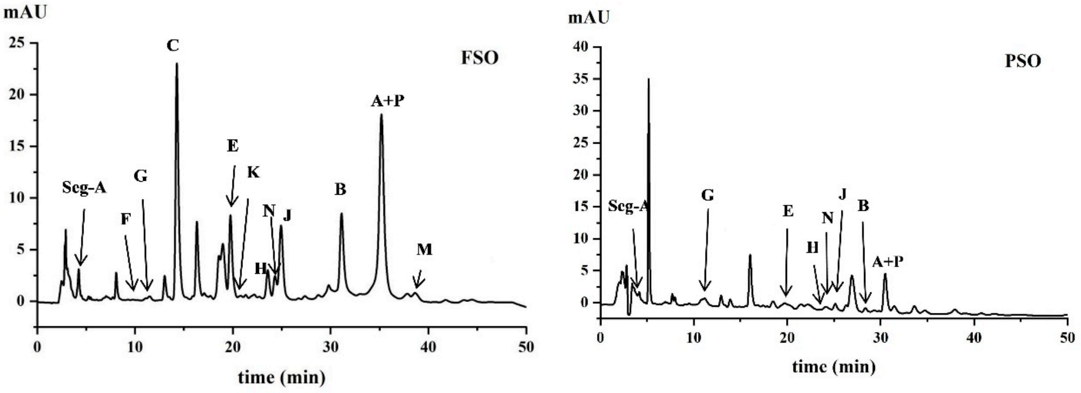

(**a**) (**b**)

Figure S5. The results of identification of cyclolinopeptides by HPLC-ESI- QTOF-MS. PSO: Perilla Seed Oil; FSO: Flaxseed Oil.

Table S2. Cyclolinopeptides in flaxseed oil detected using HPLC-QTOF-MS.

Peak No.	Retention time (min)	Cyclolinopeptides	Amino acid sequence	Chemical formula	$[M+H]^+$	Fragment ions(m/z)
1	11.886	CLF	cyclo-MsoLMsoPFFWV	$C_{55}H_{73}N_9O_{10}S_2$	1084.5017	494.4072, 450.3814
2	13.509	CLG	cyclo-MsoLMsoPFFWI	$C_{56}H_{75}N_9O_{10}S_2$	1098.5185	520.3345, 450.3815
3	16.521	CLC	cyclo-MsoLIPPFFVI	$C_{56}H_{83}N_9O_{10}S$	1074.6112	520.3345, 476.3084
4	21.187	CLE	cyclo-MsoLVFPLFI	$C_{51}H_{76}N_8O_9S$	977.5581	520.3348, 476.3086
5	21.726	CLK	cyclo-MsnLIPPFFVI	$C_{56}H_{83}N_9O_{11}S$	1090.606	977.5574, 520.335
6	25.233	CLH	cyclo-MsoLMPFFWI	$C_{56}H_{75}N_9O_9S_2$	1082.5242	682.3656, 637.3078
7	26.191	CLN	cyclo-MsoLMPFFWI	$C_{56}H_{75}N_9O_9S_2$	1082.5245	682.3655, 637.3076
8	26.509	CLJ	cyclo-MsnLVFPLFI	$C_{51}H_{76}N_8O_{10}S$	993.553	682.3656, 637.3077
9	31.589	CLB	cyclo-MLIPPFFVI	$C_{56}H_{83}N_9O_9S$	1058.6169	620.4399, 576.4135
10	37.334	CLA	cyclo-ILVPPFFLI	$C_{57}H_{89}N_9O_9$	1040.6602	961.5633, 520.3347

11	37.841	CLP	cyclo-MLMsoPFFWI	$C_{51}H_{76}N_8O_8S$	961.5433	520.3349, 476.3087
12	40.504	CLM	cyclo-MLLPFFWI	$C_{56}H_{75}N_9O_8S_2$	1066.529	968.7551, 722.5076

Table S3. Decision matrix for the improved TOPSIS.

Oils	OSI (h)	ΔSFA (%)	ΔMUFA (%)	ΔPUFA (%)	ΔAV (mg KOH/g)	ΔPOV (mmol/kg)	Δp-AV	ΔTOTOX	Δα-Tocopherol (mg/kg oil)	Δγ-Tocopherol (mg/kg oil)	ΔTocopherol (mg/kg oil)	ΔCampesterol (mg/kg oil)	Δβ-Sitosterol (mg/kg oil)	ΔStigmasterol (mg/kg oil)	ΔTotal sterol (mg/kg oil)	ΔSqualene (mg/kg oil)	ΔPolyphenols (mg GAE/kg oil)
CO	2.38	1.73	0.48	-2.20	0.07	33.62	0.95	135.43	-302.54	-653.29	-1021.36	-1856.66	-4991.58	-287.75	-7322.44	-136.41	0.80
SBO	0.8	1.59	0.80	-2.25	0.26	58.85	9.67	245.08	-273.70	-647.32	-1498.66	-369.32	-704.83	-328.87	-1403.02	-121.99	0.87
FSO	0.12	0.69	1.73	-2.42	0.24	49.26	21.39	218.44	0.00	-235.79	-235.79	-132.82	-325.20	-138.12	-1014.14	-432.34	0.41
PSO	0.07	0.79	1.54	-2.34	0.66	36.50	27.40	173.38	0.00	-66.67	-66.67	-138.06	-1213.21	-40.94	-1611.35	-193.48	0.22
SO	3.83	0.89	0.14	-1.03	0.54	32.89	1.59	133.16	0.00	-652.52	-652.52	-105.60	-195.39	-74.22	-446.48	-350.36	12.26
PNO	3.78	0.08	1.01	-1.09	0.13	56.15	2.24	226.85	-710.32	-110.26	-948.17	-73.34	-206.35	-229.84	-509.53	-353.50	6.88
RO	4.06	1.17	0.00	-1.17	0.15	41.79	7.52	174.68	-686.02	-238.02	-993.92	-247.21	-493.42	0.00	-1091.95	-212.61	5.07
POL	6.04	1.72	-0.43	-1.14	0.15	75.21	2.22	303.04	-368.59	-357.37	-1640.28	-187.95	-439.52	-175.24	-802.71	-196.87	1.113
CMO	1.78	1.14	-0.69	-0.46	0.81	20.23	2.25	83.16	-283.95	0.00	-283.95	-176.79	0.00	0.00	-3729.76	-248.26	0.71
HSSO	2.13	2.87	-1.38	-1.49	0.02	15.41	0.15	61.80	-1290.24	0.00	-1311.74	-308.46	-1025.27	-140.01	-1473.74	-63.34	0.83

Note: OSI: oxidative stability index; Δ: Increment of data; PLO: Palm olein; CO: Corn Oil; PSO: Perilla Seed Oil; PNO: Peanut Oil; CMO: Camellia seed Oil; RO: Rapeseed oil; SBO: Soybean Oil; HSSO: High Oleic Acid Sunflower Seed Oil; FSO: Flaxseed Oil; SO: Sesame Oil.
